# Urban–sub-urban–rural variation in the supply and demand of emergency medical services

**DOI:** 10.3389/fpubh.2022.1064385

**Published:** 2023-01-25

**Authors:** Yue Li, Ji Li, Jiayu Geng, Tao Liu, Xin Liu, Haojun Fan, Chunxia Cao

**Affiliations:** ^1^Wenzhou Safety (Emergency) Institute, Tianjin University, Wenzhou, China; ^2^School of Public Administration, College of Management and Economics, Tianjin University, Tianjin, China; ^3^Institute of Disaster and Emergency Medicine, Tianjin University, Tianjin, China; ^4^Emergency Department, Tianjin Medical Emergency Center, Tianjin, China

**Keywords:** emergency medical services, supply and demand matching, healthcare reform, healthcare resources, megacity

## Abstract

**Background:**

Emergency medical services (EMSs) are a critical component of health systems, often serving as the first point of contact for patients. Understanding EMS supply and demand is necessary to meet growing demand and improve service quality. Nevertheless, it remains unclear whether the EMS supply matches the demand after the 2016 healthcare reform in China. Our objective was to comprehensively investigate EMS supply–demand matching, particularly among urban vs. sub-urban vs. rural areas.

**Methods:**

Data were extracted from the Tianjin Medical Priority Dispatch System (2017–2021). From supply and demand perspectives, EMS resources and patient characteristics were analyzed. First, we performed a descriptive analysis of characteristics, used Moran's I to explore the spatial layout, and used the Gini coefficient to evaluate the equity of EMS supply and demand. Second, we analyzed urban–sub-urban–rural variation in the characteristics of EMS supply and demand by using the chi-square test. Finally, we examined the association between the EMS health resource density index and the number of patients by using the Spearman correlation and divided supply–demand matching types into four types.

**Results:**

In 2021, the numbers of medical emergency stations and ambulances were 1.602 and 3.270 per 100,000 population in Tianjin, respectively. There were gradients in the health resource density index of the number of emergency stations (0.260 vs. 0.059 vs. 0.036; *P* = 0.000) in urban, sub-urban, and rural areas. There was no spatial autocorrelation among medical emergency stations, of which the G values by population, geographical distribution, and the health resource density index were 0.132, 0.649, and 0.473, respectively. EMS demand was the highest in urban areas, followed by sub-urban and rural areas (24.671 vs. 15.081 vs. 3.210 per 1,000 population and per year; *P* = 0.000). The EMS supply met the demand in most districts (*r* = 0.701, *P* = 0.003). The high supply–high demand types with stationary demand trends were distributed in urban areas; the low supply–high demand types with significant demand growth trends were distributed in sub-urban areas; and the low supply–low demand types with the highest speed of demand growth were distributed in rural areas.

**Conclusion:**

EMS supply quantity and quality were promoted, and the supply met the demand after the 2016 healthcare reform in Tianjin. There was urban–sub-urban–rural variation in EMS supply and demand patterns.

## 1. Introduction

### 1.1. Background

Emergency medical services (EMSs) are integral to immediate critical care, stabilization of life-threatening diseases, timely triage, and transportation in prehospital settings (1–3). Most countries attach great importance to meeting EMS demand, and comprehensive healthcare reform policies have been initiated successively by national legislatures in several countries (4–10). However, EMSs are still mainly experiencing supply–demand shortages and imbalances. The available wealth has not been distributed evenly in different regions, leading to a widening gap in health outcomes, resource supply, and demand for health services in urban, sub-urban, and rural areas (11–13). Furthermore, during the coronavirus disease 2019 (COVID-19) pandemic, EMS activations for cardiac events and opioid use/overdose are increasing, and ambulance response times are deteriorating (14, 15). The supply and demand of EMS represent a challenge for both the EMS and hospital-based emergency departments (16, 17).

Most research has focused mainly on the spatial layout and accessibility of EMS facilities for optimal allocation (18–23). Liu et al. found that the equity of supply and demand for EMS resources is low based on data from death surveillance from 2008 to 2012 in Chongqing, China (24). As more investigations have begun to focus on rural areas, it is reported that access to healthcare and treatment resources in rural areas is limited (25). Urban–sub-urban–rural disparities in EMS supply have become a current crucial public health issue (26). To address this limitation, the Outline of Healthy China 2030 Plan highlighted that we should improve the medical and health service system from both the supply and demand sides in October 2016 (27). China has set up reform pilot programs of purely prehospital care models in several cities (i.e., Tianjin, Shanghai, Nanjing, Wuhan, and Hangzhou) to further deepen the reform of the medicine and healthcare system. Tianjin implemented comprehensive healthcare reform to improve equity and efficiency by adjusting EMS models in 2017. Since then, Tianjin has employed the purely prehospital care model comprising mainly prehospital emergency services with no sickbeds, and electronic medical records were standardized based on the Medical Priority Dispatch System (MPDS) in Tianjin to standardize EMS patient care reporting and facilitate the collection of data. Nevertheless, EMS supply–demand matching (SDM) has not been comprehensively investigated after the 2016 healthcare reform, particularly in relation to urban vs. sub-urban vs. rural areas. Nevertheless, EMS supply–demand matching (SDM) has not been comprehensively investigated after the 2016 healthcare reform, particularly in relation to urban, sub-urban vs. rural.

### 1.2. Goals of investigation

The purpose of this study was to comprehensively determine urban–sub-urban–rural variation in the supply and demand patterns of EMS by examining the characteristics, spatial layout, and equity after healthcare reform in the megacity of Tianjin, China. The results will provide a reference for further advancing medical reform, implementing the Healthy China 2030 strategy, and proposing feasible proposals to improve EMS sustainable development in the megacities of developing countries.

## 2. Methods

### 2.1. Study setting

Tianjin is a coastal megacity, administratively equivalent to a province, in the North China Plain, with a population of approximately 14 million people and an area of 11,947 km^2^. Tianjin has a single EMS provider, namely, the Tianjin Medical Emergency Center, operated by the Tianjin Health Commission; the Tianjin Medical Emergency Center is a publicly funded system and covers all emergency medical calls within the city. In 2017, focused healthcare reform was initiated in Tianjin to set up a pilot program for a purely prehospital care model, and the electronic medical records and intelligent dispatch were standardized based on the Medical Priority Dispatch System (MPDS). An electronic record was automatically created for each patient by using EMS with a unique registration number and was recorded in real-time on the operating system. The database contained a complete collection of every EMS dispatch record generated in all districts of Tianjin.

We designed a retrospective study to comprehensively evaluate the supply and demand patterns of EMS after the healthcare reform in the urban, sub-urban, and rural areas of Tianjin.

### 2.2. Data sources

Data related to EMS supply and demand during the calendar years 2017 through 2021 were extracted based on routinely collected ambulance dispatch data from the MPDS of Tianjin Emergency Medical Center. The data elements about both EMS supply and demand in this study were as follows: (i) the numbers of medical emergency stations, ambulances, prehospital emergency physicians (PEPs), and nurses; and (ii) the date of the emergency call, demographic characteristics and initial diagnosis of each patient, and EMS incident location. The inclusion criteria were as follows: patients transported by ambulance from the scene to the hospital or from one hospital to another hospital. Data related to the current situation were from the Tianjin Statistical Yearbook and Health Statistical Yearbook.

### 2.3. Variables and definitions

#### 2.3.1. Classification of regions

We analyzed the variation in EMS demand in urban, sub-urban, and rural areas according to EMS incident location. Tianjin consists of 16 districts. Based on the geocoding system, the districts were divided into three regions: urban areas (Heping, Hexi, Hedong, Nankai, Hebei, and Hongqiao districts), sub-urban areas (Dongli, Xiqing, Jinnan, and Beichen districts), and rural areas (Baodi, Wuqing, Ninghe, Jinghai, Jixian, and Binhai districts).

#### 2.3.2. Classification of disease categories

Prehospital emergency physicians made initial diagnoses and recorded electronic records of each patient according to the MPDS (Supplementary Table 1). The sophisticated PEPs regularly reviewed the accuracy of the diagnoses and the quality of the records that day.

### 2.4. Spatial analysis

#### 2.4.1. Global Moran's I and Anselin Local Moran's I

Moran's I is a way to measure spatial autocorrelation. Global Moran's I was used for the spatial autocorrelation of EMS supply (28, 29). Evidence of local cluster characteristics was evaluated using Anselin Local Moran's I. These indices were calculated as follows:


(1) $$Gobal\;Moran^{\prime }s\;I=\frac{n\sum_{i=1}^{n}\sum_{j=1}^{n}W_{ij}(Y_{i}-\bar{Y})(Y_{j}-\bar{Y})}{\sum_{i\neq j}W_{ij}{\left(Y_{i}-\bar{Y}\right)}^{2}}$$

(2) $$Local\;Moran^{\prime }s\;I=\frac{\left(Y_{j}-\bar{Y}\right)}{\sum_{i=1}^{n}{\left(Y_{j}-\bar{Y}\right)}^{2}/(n-1)}\;*\;\sum_{j\neq i}^{n}W_{ij}(Y_{j}-\bar{Y})$$

where *i* and *j* are the region indices and *W*_*ij*_ indicates the adjacency between area *i* and area *j*. Two districts were considered adjacent if they shared a border. *Y*_*i*_ and *Y*_*j*_ denote the numbers of medical emergency stations in areas *i* and *j*, respectively, and *Y* is the average of the number of medical emergency stations in the entire district. A value of 0 indicates that the data have no spatial autocorrelation. A positive Moran's I indicates the clustering of similar values, whereas a negative Moran's I indicates the clustering of dissimilar values.

#### 2.4.2. Gini co-efficient and health resource density index

The Gini coefficient is a tool used to assess the equity of health resource supply and demand in terms of demographic and geographical aspects. The Gini coefficients were derived from the Lorenz curves. With regard to the Lorenz curve, the x-axis represents the cumulative percentage of population or geography, and the y-axis represents the cumulative percentage of EMS supply and demand. A 45° line indicates absolute equity. A larger distance from the diagonal line indicates greater unfairness. Formula (3) was used to calculate the Gini coefficients.


(3) $$G=1-\sum {}_{i=0}^{k-1}(Y_{i}+1+Y_{i})(X_{i}+1-X_{i})$$

*X*_*i*_ is the cumulative percentage of health resources and demand in the *i*th district of Tianjin city after ranking according to the per capita or the regional average share of health resources and demand. *Y*_*i*_ is the cumulative percentage of population and geography in the *i*th district of Tianjin after ranking according to the per capita and the regional average share of EMS supply and demand. *K* is the total number of districts. *G* is the value of the Gini coefficient.

The health resource density index (HRDI) displays the influence of population and geographical factors on the agglomeration of EMS supply and demand while avoiding bias caused by a single population or geographical aspect. Formula (4) was used to calculate the HRDI.


(4) $$HRDI=\frac{HR_{i}}{\sqrt{A_{i}P_{i}}}$$

*HR*_*i*_ is the health resource and demand quantity for the *i*th district. *A*_*i*_ is the geography of the *i*th district (km^2^). *P*_*i*_ is the population of the *i*th district. *HRDI* is the value of the HRDI.

### 2.5. Statistical analysis

In the analysis of EMS supply, the following data were analyzed: the number of medical emergency stations, ambulances, PEPs, and nurses with consideration of demographic and geographical dimensions. In the analysis of EMS demand, the date of the emergency call, gender, initial diagnosis of each patient, and EMS incident location was analyzed.

Descriptive analysis of the EMS supply and demand characteristics was expressed as the median and interquartile spacing [M (Q1, Q3)]. Categorical variables were analyzed by using the chi-square test, and continuous variables with a near-normal distribution and variables with skewed distributions were analyzed by using the *t*-test and the Mann–Whitney test, respectively. Joinpoint regression was used to identify annual potential trends in the EMS demand in Tianjin from 2017 to 2021 by calculating the annual percentage change (APC). Counts from the Poisson regression model and grid search method were selected. A permutation test with the Bayesian information criterion was used to select the best model, applying a Bonferroni correction to the type I error to correct for multiple testing. The APC and 95% confidence intervals (CI) were estimated every year on a log scale. Associations between basic characteristics of the current situation and EMS demand and EMS resource supply and demand in different districts were examined by using the Spearman correlation.

We divided the districts into two groups (high EMS supply and low EMS supply) according to the median value of the HRDI and divided the districts into two groups (high EMS demand and low EMS demand) according to the average number of EMS patients per year. Relying on the aforementioned, we divided the supply and demand patterns of EMS into four types: high supply–high demand, high supply–low demand, low supply–low demand, and low supply–high demand.

Data management was performed using Microsoft Excel 2019 (Microsoft Corp, Redmond, WA), and statistical analyses were carried out using the Statistic Package for Social Sciences, version 26.0 (IBM Corp, Armonk, New York). Geospatial visualizations and spatial analysis were obtained using ArcGIS 10 (ESRI, Redlands, CA). The Joinpoint Regression Program, version 4.9.0.0 (Statistical Research and Applications Branch, National Cancer Institute), was used to carry out joinpoint regression (30, 31). The estimate was considered to be statistically significant at α = 0.05, and tests were two-tailed.

## 3. Results

### 3.1. Urban–sub-urban–rural variation in EMS supply

#### 3.1.1. Characteristics of EMS supply

**Figure 1:**
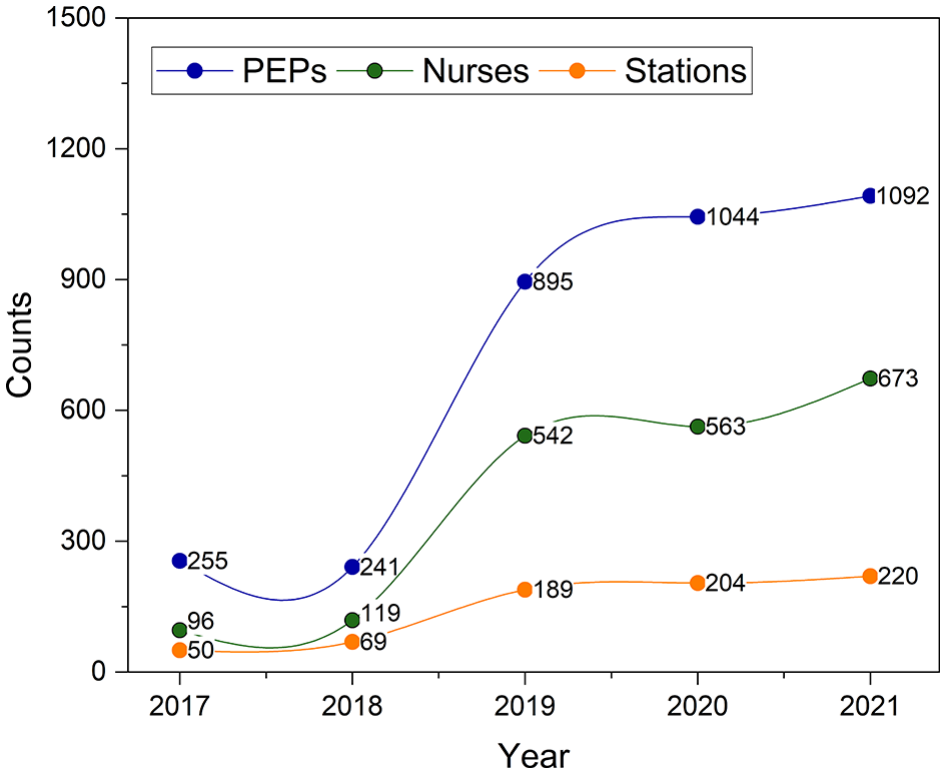
Number of medical emergency stations, PEPs, and nurses in Tianjin. PEPs, prehospital emergency physicians.

The purely pre-hospital care model was set up with the three-level network system of “medical emergency center, medical emergency sub-centers, and medical emergency stations” in Tianjin in 2017. The number of EMS resources, such as medical emergency stations, PEPs, and nurses, greatly increased from 2017 to 2021, especially in 2019 (Figure 1).

As of 2021, there was one medical emergency center, 11 medical emergency subcenters, and 220 medical emergency stations. The number of medical emergency stations was 1.602 per 100,000 population (approximately one medical emergency station per 60,000 population).

The number of ambulances was 449 and was 3.270 per 100,000 population (approximately 1 ambulance per 30,000 population) in 2021. There were four types of ambulances as follows: type 1 ambulances (with oxygen delivery facility and for nonemergency patients), type 2 ambulances (with basic electrocardiograph monitoring, defibrillation integrated machine, electrocardiogram, blood glucose meter, essential drug, oxygen delivery facility, and for most emergency patients), type 3 ambulances (in addition to having basic equipment, these ambulances were equipped with a ventilator, a chest compression device, an infusion pump, and syringe pump, and were for the most severe or critical patients), and type 4 ambulances (i.e., negative-pressure ambulances; these ambulances were equipped with an in-vehicle negative-pressure purification system and were used for transporting patients confirmed of COVID-19, suspected patients, and their close contacts).

Upon each activation, each ambulance was equipped with a PEP, a nurse, two stretchers, and a driver. As of 2021, there were 1,092 PEPs and 673 nurses. The doctor–nurse ratio was 1.624:1. The number of PEPs was 0.795 per 10,000 population.

#### 3.1.2. Spatial layout of EMS supply

**Figure 2:**
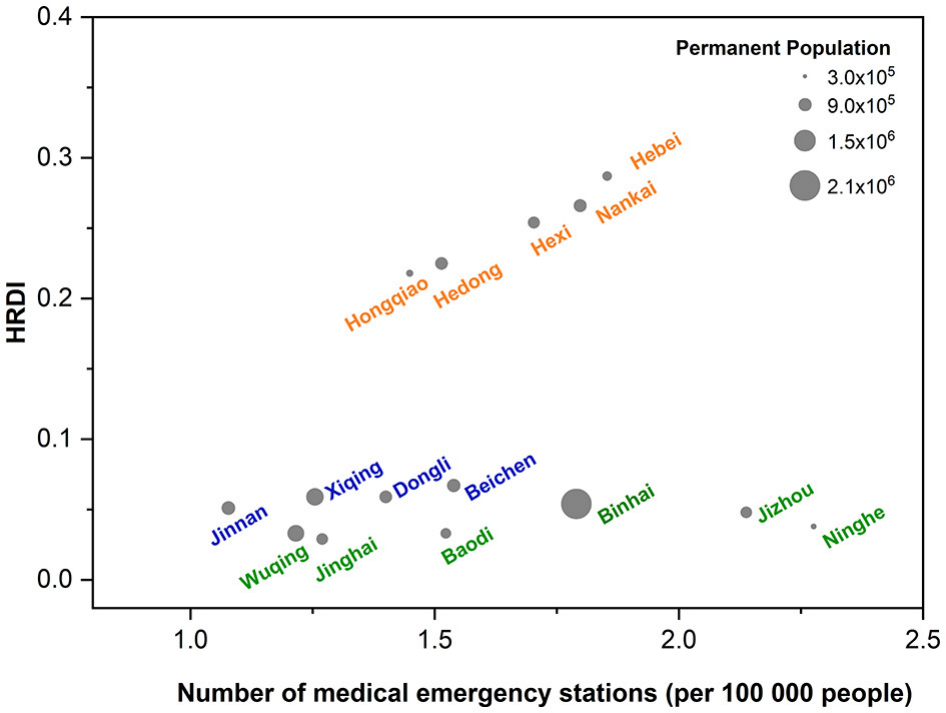
Equity in EMS supply in Tianjin. HRDI, health resource density index. The orange font represents urban areas (Heping, Hexi, Hedong, Nankai, Hebei, and Hongqiao districts); the blue font represents sub-urban areas (Dongli, Xiqing, Jinnan, and Beichen districts); and the green font represents rural areas (Baodi, Wuqing, Ninghe, Jinghai, Jixian, and Binhai districts).

Supplementary Table 2 summarizes the development state and EMS supply of each district in Tianjin in 2021. Most districts accorded with the standard of 1.216–2.535 emergency stations per 100,000 population. In terms of population distribution, the number of emergency stations was the highest in urban areas, followed by rural and sub-urban areas (1.809 vs. 1.132 vs. 1.170 per 100,000 population; *P* = 0.000). In terms of geographical distribution (0.463 vs. 0.026 vs. 0.009 per 1 km^2^; *P* = 0.000) and the HRDI (0.288 vs. 0.059 vs. 0.039; *P* = 0.000), the number of emergency stations was the highest in urban areas, followed by sub-urban and rural areas (Figure 2). Notably, there were gradients in the HRDI of districts (urban areas: 0.260 [0.223, 0.335]; sub-urban areas: 0.059 [0.053, 0.065]; and rural areas: 0.036 [0.032, 0.050]). The number of medical emergency stations had no spatial autocorrelation (Moran's I = −0.230, *Z* = −1.759, *P* = 0.0786). The high-low cluster area was in Binhai District; the low-high cluster area was in Jinghai District. The number of medical emergency stations per 100,000 population was the highest in Heping District and the lowest in Jinghai District. The number of medical emergency stations per 1 km^2^ was the highest in Heping District and the lowest in Ninghe District. The HRDI with medical emergency stations was the highest in Heping District and the lowest in Wuqing District.

#### 3.1.3. Equity of EMS supply

Considering demographic and geographical dimensions, we analyzed three aspects of equity in the distribution of EMS resources. The G values of EMS supply by population were 0.132 in Tianjin, thus indicating that the EMS supply by population distribution in Tianjin was relatively fair. The G values of EMS supply by geographical distribution and HRDI in Tianjin were 0.649 and 0.473, respectively, thus indicating that the EMS supply by geographical distribution and HRDI distribution in Tianjin was relatively unfair.

### 3.2. Urban–sub-urban–rural variation in EMS demand

#### 3.2.1. Characteristics of EMS demand

**Figure 3:**
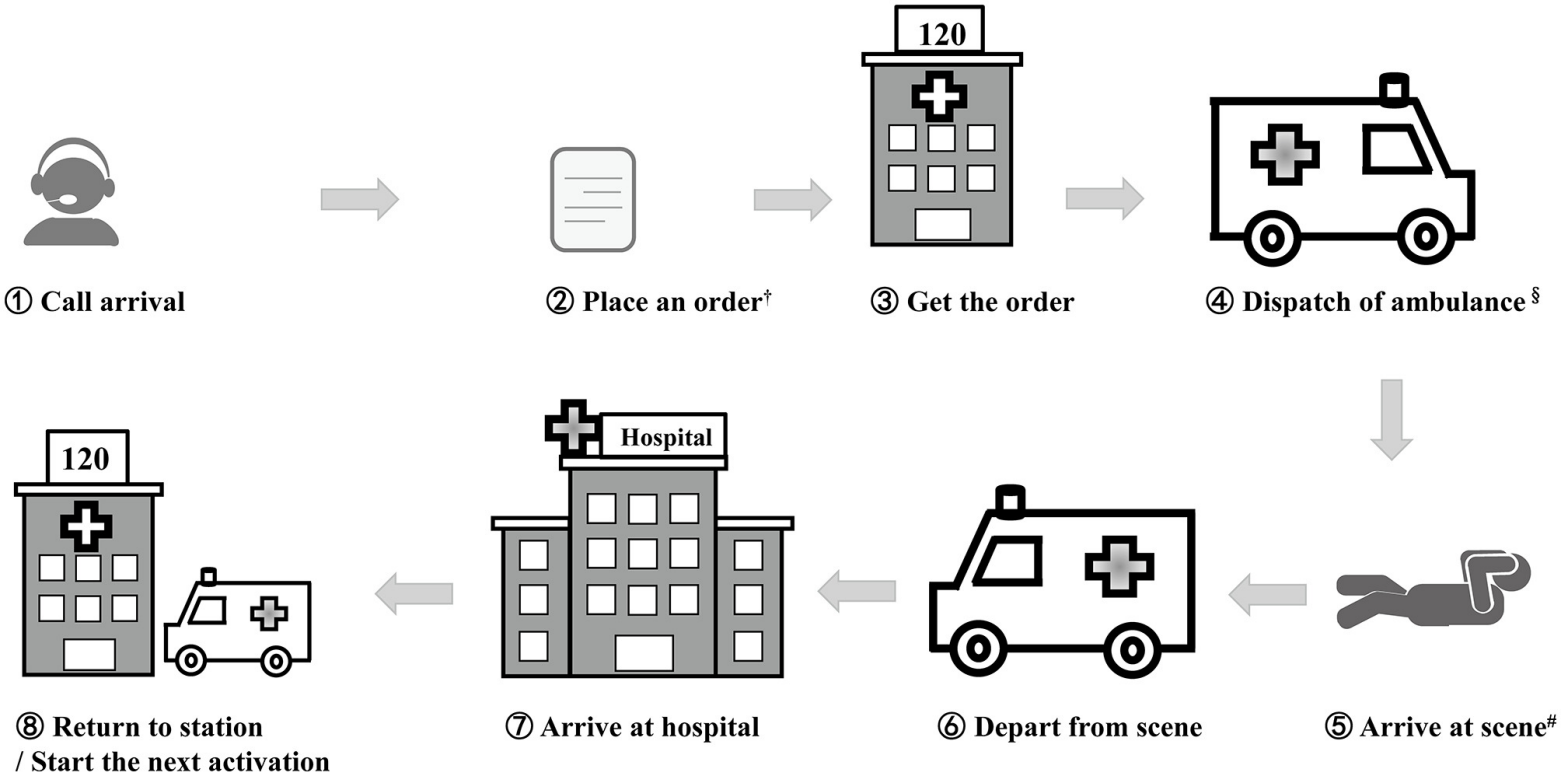
Process of EMS utilization in Tianjin. ^†^Step 1 and Step 2 were implemented simultaneously in 2021. ^§^The ambulances were directly dispatched by the municipal emergency medical center in urban and sub-urban areas; the rural implemented regional ambulances dispatched by the emergency medical subcenter. ^**#**^Some EMS utilization was transportation from one hospital. Response time = ⑤-①.

The process of EMS utilization in Tianjin was as follows (Figure 3): The call arrives, an order is placed, the order is received, an ambulance is dispatched, the ambulance crew arrives at the scene, the ambulance crew departs from the scene, the ambulance crew arrives at the hospital, and the ambulance crew returns to the station or starts the next activation. EMS utilization involves transportation from one hospital to another. Any request for prehospital emergency medical assistance was made through the national emergency telephone number (120). The municipal emergency medical center uniformly recorded all calls and managed all activations by using the MPDS in Tianjin since 2017. The ambulances were directly dispatched by the municipal emergency medical center in urban and sub-urban areas. Regional ambulances were dispatched in rural areas by the emergency medical sub-center.

The percentage of prehospital emergency calls received within 10 seconds (s) was 76% in 2017, while the percentage was maintained at 100% from 2019 to 2021 (*P* = 0.000) (Supplementary Figure 1). Steps 1 to 2 took approximately 1 min before 2021. Steps 1 and 2 were implemented simultaneously starting in 2021. The response time was from the time of call arrival to the time of arrival at the scene. From 2017 to 2021, the response time continued to decrease, from 13.300 min to 7.863 min.

From 2017 to 2021, 1,010,771 patients using EMS were included in this study, and 202,154 patients per year were treated in Tianjin. The EMS demand increased with an annual growth rate of 33.6 (95% CI 15.0, 55.2) (Supplementary Figure 1). The EMS demand rate increased from 5.702 patients in 2017 to 23.064 patients per year and 1,000 people in Tianjin. The fatality rate decreased from 4.774% in 2017 to 2.921% in 2021 (*P* = 0.000).

**Figure 4:**
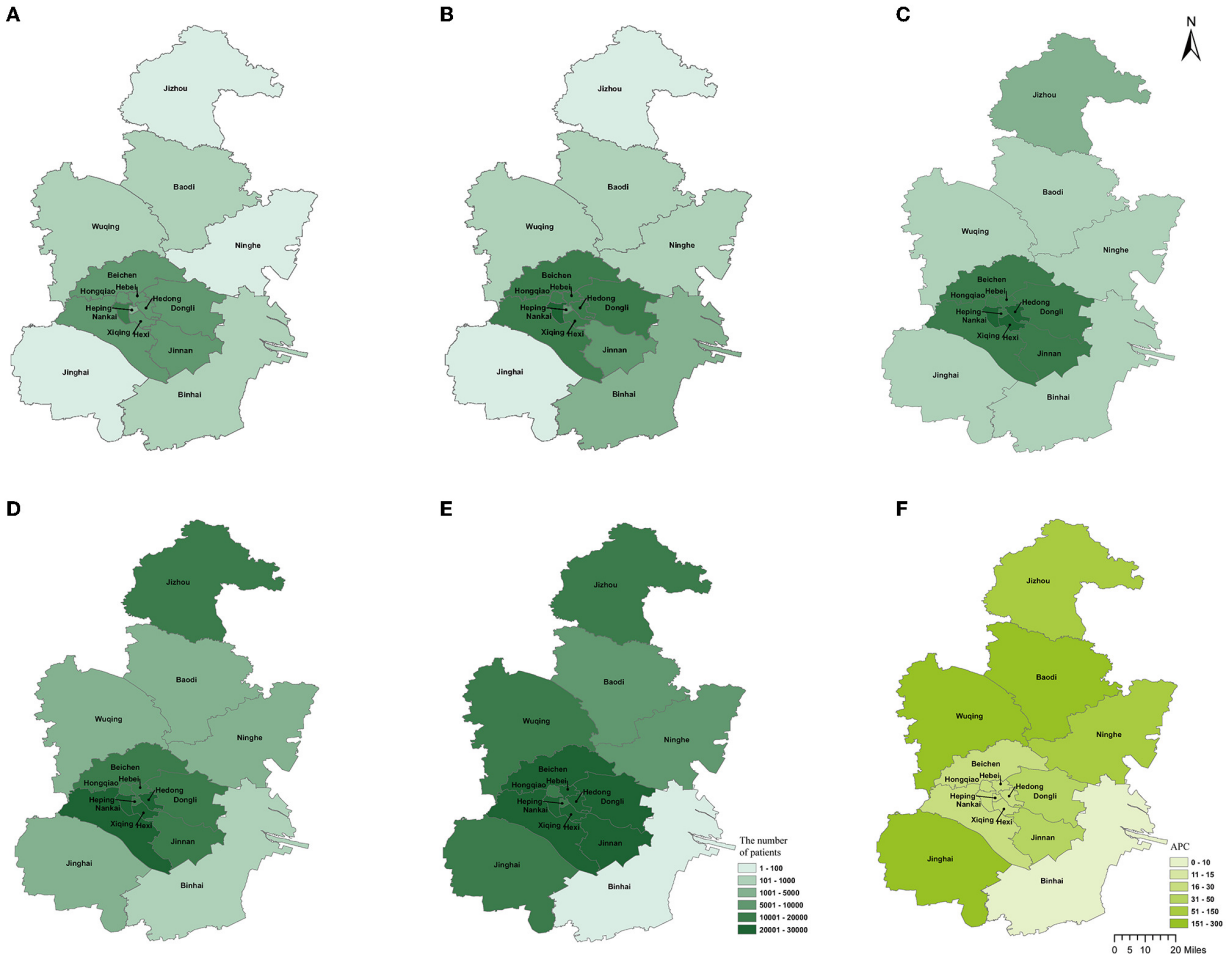
Geographical distribution and APC of EMS patients in Tianjin. **(A)** The number of patients using EMS in 2017; **(B)** the number of patients using EMS in 2018; **(C)** the number of patients using EMS in 2019; **(D)** the number of patients using EMS in 2020; **(E)** the number of patients using EMS in 2021; **(F)** APC of EMS patients in Tianjin from 2017 to 2021.

The joinpoint analysis indicated that annually from 2017 to 2021, the numbers of EMS patients in sub-urban (APC = 31.1, 95% CI 8.2, 58.8) and rural areas (APC = 164.7, 95% CI 63.6, 328.4) increased significantly, while the number of EMS patients in urban areas was stationary (APC = 17.9, 95% CI −6.9, 49.3). Overall trends for increased EMS patient numbers in most sub-urban and rural areas, including Beichen (APC = 29.6, 95% CI 6.8, 57.1), Dongli (APC = 30.6, 95% CI 9.4, 56.0), Xiqing (APC = 27.3, 95% CI 2.2, 58.4), Jinnan (APC = 37.6, 95% CI 12.7, 68.0), Wuqing (APC = 251.8, 95% CI 112.5, 482.2), Ninghe (APC = 124.4, 95% CI 61.2, 212.4), Baodi (APC = 184.6, 95% CI 74.7, 363.7), and Jinghai (APC = 195.7, 95% CI 55.0, 463.9) districts, were observed in detail. The number of EMS patients in other districts remained stable from 2017 to 2021 (Figure 4).

The male-to-female ratio of patients using EMS in Tianjin from 2017 to 2021 was 1.116:1, 1.298:1, and 1.323:1 in the urban, sub-urban, and rural areas, respectively (*P* = 0.000). There was a greater demand for EMS among male patients (*P* = 0.000). An age difference was found among urban, sub-urban, and rural patients using EMS; urban EMS patients were older (*P* = 0.000) (Supplementary Figure 2).

In urban areas, most diagnoses were non-specific diagnoses (35,308, 58.341%), followed by diagnoses of traumatic injury (14,951, 46.595%), cerebrovascular diseases (7,743, 53.497%), cardiovascular diseases (7,340, 55.608%), and respiratory diseases (6,217, 58.136%). In sub-urban areas, most diagnoses were non-specific diagnoses (20,883, 34.505), followed by diagnoses of traumatic injury (11,737, 36.580%), cerebrovascular diseases (5,176, 35.757%), cardiovascular diseases (4,193, 31.763), and respiratory diseases (3,553, 33.222%). Traumatic injury (5,399, 16.825%) was the most common diagnosis in rural areas; the next most common were non-specific diagnoses (4,330, 7.154%) and diagnoses of cardiovascular diseases (1,667, 12.629), cerebrovascular diseases (1,555, 10.746%), and respiratory diseases (924, 8.642%). Note that 71.418% of patients with genitourinary system diseases in Tianjin were in urban areas; 52.735% of patients with infectious diseases in Tianjin were in sub-urban areas. Compared to patients with other diseases, traumatic injury and chemical–physical damage patients seemed to be more numerous in rural areas (Table 1).

**Table 1:** Disease spectrum of patients using EMS in urban, sub-urban, and rural of Tianjin, China, from 2017 to 2021.

**Diseases**	**Urban, No. (%)**	**Sub-urban, No. (%)**	**Rural, No. (%)**	**Total**
Non-specific diagnoses	35,308 (58.341)	20,883 (34.505)	4,330 (7.154)	60,520
Traumatic injury	14,951 (46.595)	11,737 (36.580)	5,399 (16.825)	32,087
Cerebrovascular diseases	7,743 (53.497)	5,176 (35.757)	1,555 (10.746)	14,474
Cardiovascular diseases	7,340 (55.608)	4,193 (31.763)	1,667 (12.629)	13,200
Respiratory diseases	6,217 (58.136)	3,553 (33.222)	924 (8.642)	10,695
Digestive system diseases	4,070 (61.729)	1,964 (29.795)	559 (8.476)	6,593
Poisoning	1,931 (52.422)	1,252 (33.981)	501 (13.597)	3,683
Tumor	1,840 (66.135)	802 (28.834)	140 (5.031)	2,783
Genitourinary system diseases	1,750 (71.418)	583 (23.798)	117 (4.784)	2,450
Other neurological disorders	1,231 (51.049)	819 (33.941)	362 (15.010)	2,412
Exhaustion	1,496 (63.185)	713 (30.139)	158 (6.675)	2,367
Obstetrics and gynecology diseases	914 (51.337)	751 (42.191)	115 (6.472)	1,780
Endocrine metabolic diseases	650 (60.197)	323 (29.904)	107 (9.900)	1,079
Musculoskeletal and connective tissue disorders	395 (64.123)	167 (27.045)	54 (8.831)	616
Ear nose and throat and ophthalmology diseases	376 (61.708)	192 (31.461)	42 (6.831)	609
Pediatric diseases	363 (59.993)	193 (31.946)	49 (8.061)	605
Mental and behavioral disorders	282 (54.864)	195 (37.938)	37 (7.198)	514
Infectious diseases	181 (42.403)	226 (52.735)	21 (4.862)	428
Chemical-physical damage	194 (45.950)	156 (36.902)	73 (17.148)	422
Blood diseases	277 (69.350)	92 (23.100)	30 (7.550)	400
Skin diseases	173 (61.963)	74 (26.433)	32 (11.605)	279
Shock	110 (49.194)	74 (33.065)	40 (17.742)	223
Fluid and electrolyte imbalance	117 (53.529)	67 (30.522)	35 (15.949)	218
Immune system diseases	60 (49.338)	40 (33.444)	21 (17.219)	

#### 3.2.2. Spatial layout of EMS demand

Urban areas had the most demand for EMS (100,095 patients per year); sub-urban areas (58,663 patients per year) and rural areas (18,99 patients per year) had the next highest demand (24.671 vs. 15.081 vs. 3.210 per 1,000 population and per year; *P* = 0.000). Spatially, the demand for EMS in Tianjin was high in the middle and low on the sides (Figure 4). The area with the most patients using EMS was Nankai District (22 576, 12.700%), and the area with the fewest was Binhai District (656, 0.369%). The EMS demand rate per year and 1,000 population were higher in Heping (26.320), Hebei (26.224), and Hexi (25.083) districts and lower in Binhai (0.318), Wuqing (3.249), and Baodi (3.442) districts. The Spearman correlation revealed that EMS demand was positively associated with population density (*r* = 0.882, *P* = 0.000) and population aged over 60 (*r* = 0.571, *P* = 0.021). EMS demand was not associated with the gross domestic product per capita (*r* = −0.035, *P* = 0.897).

#### 3.2.3. Equity of EMS demand

Considering demographic and geographical dimensions, we also analyzed the three aspects of equity in EMS demand (Supplementary Table 3). From 2017 to 2021, the G values of the EMS supply by population, geographical distribution, and HRDI gradually decreased in Tianjin. The annual trend of EMS supply G values by population (APC = −15.7, 95% CI −26.7, −3.0), geographical distribution (APC = −0.9, 95% CI −1.2, −0.7), and HRDI (APC = −3.6, 95% CI −5.6, −1.5) in Tianjin decreased significantly.

### 3.3. Urban–sub-urban–rural variation in SDM patterns

**Figure 5:**
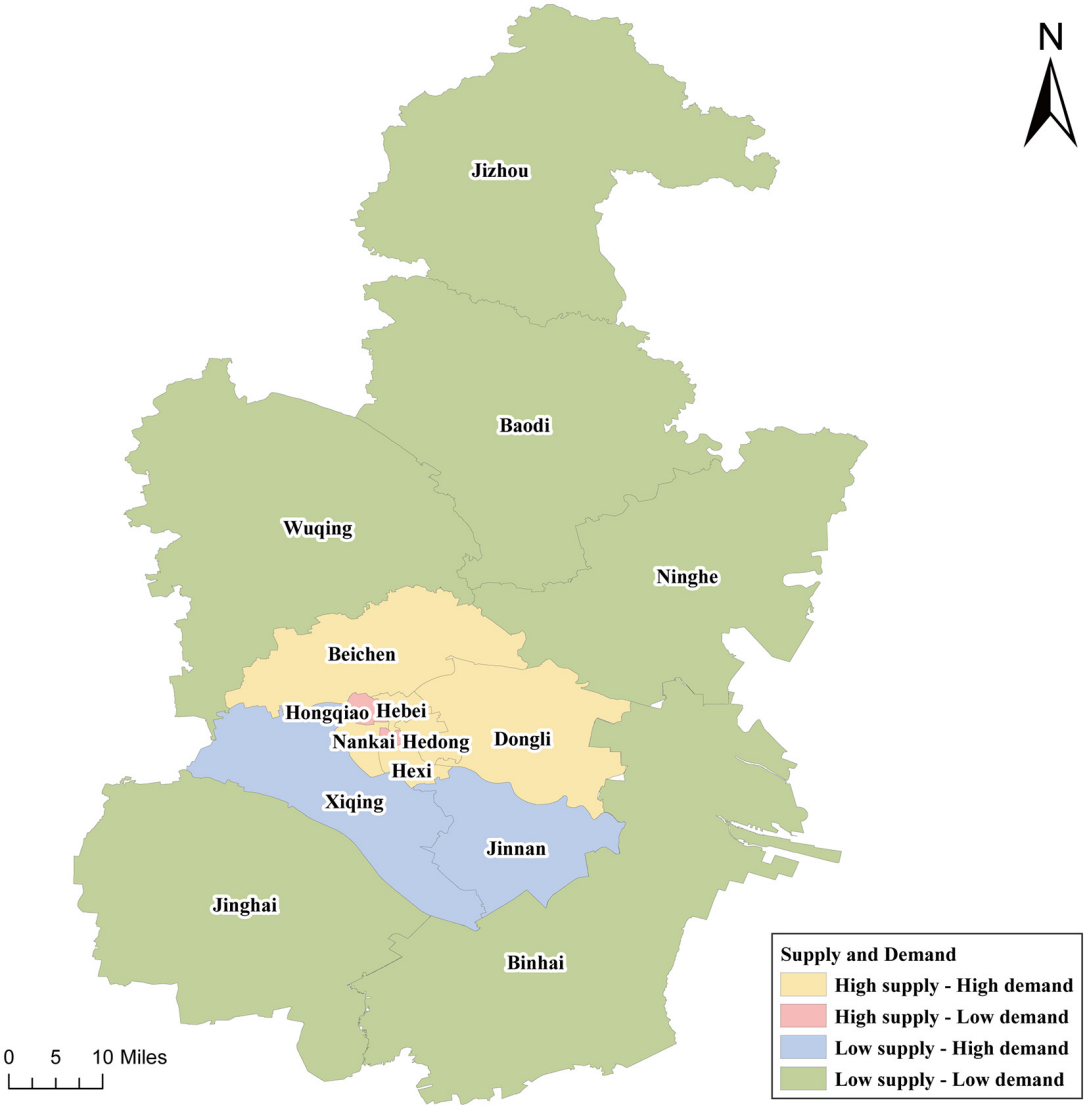
Supply–demand matching pattern of EMS at different levels in Tianjin. Urban areas include Heping, Hexi, Hedong, Nankai, Hebei, and Hongqiao districts. Sub-urban areas include Dongli, Xiqing, Jinnan, and Beichen districts. Rural areas include Baodi, Wuqing, Ninghe, Jinghai, Jixian, and Binhai districts.

There was a positive association between the HRDI of medical emergency stations and the number of EMS patients (*r* = 0.701, *P* = 0.003). The matching types of EMS supply and demand were mainly “high supply–high demand” in urban areas (Nankai, Hexi, Hedong, and Hebei districts) and in sub-urban areas (Beichen and Dongli districts) and “low supply–low demand” in rural areas (Jizhou, Wuqing, Jinghai, Baodi, Ninghe, and Binhai districts) (Figure 5). There were a few “high supply–low demand” districts in urban areas (Hongqiao and Heping districts) and “low supply–high demand” districts in sub-urban areas (Xiqing and Jinnan districts). In general, most of the urban areas were high supply–high demand areas, and most of the rural areas were low supply–low demand areas.

## 4. Discussion

### 4.1. Characteristics of EMS supply and demand

Emergency medical services in Tianjin are still developing. EMS resources, including the number of medical emergency stations, ambulances, and human resources, increased after years of reform and development. The number of medical emergency stations was approximately 1 per 60,000 population in Tianjin in 2021; this result agreed with the criterion of the plan that stipulates the standard of 1 per 80,000 population and more than 1 per 140,000 population in Shanghai (4). The number of ambulances was approximately 1 per 30,000 population in Tianjin in 2021; this result agreed with guidance on further improving EMS in China in 2020. It has been reported that the number of ambulances was 1 per 30,000 population in Beijing and 1 per 40,000 population in Shanghai. In developed countries, the number of ambulances per capita seems to be higher; for example, the number of ambulances per capita is 1 per 30,000 population in Turkey and 1 per 4,350 population in Australia (32, 33).

Moreover, EMS quality was promoted after healthcare reform based on the data on prehospital emergency calls received within 10 s, the response time, and the fatality rate. The percentage of prehospital emergency calls received within 10 s has become an important quality control indicator for EMS. Since the healthcare reform in 2017, all emergency calls have been answered by the medical emergency center, and the number of call takers has increased. The percentage of prehospital emergency calls received within 10 s has been maintained at 100% since 2019. The response time continued to decrease to 7.863 min in Tianjin in 2021. It has been reported that Shanghai required the average EMS response time to be within 12 min (4), the median EMS response time was 9.12 min in 2017 in Shenzhen (34), and the EMS response time was approximately 6.4 min in Japan (35).

We found that the EMS demand per 1,000 population was 14.580 each year in Tianjin, which, compared to other places, seems to have a lower demand for EMS. It has been reported that EMS demand per 1,000 population is 23.9 each year in Taiwan and China and 31–48 in America (36). Our results indicated that the EMS demand would continue to grow over the following years in Tianjin. In particular, population increase and economic growth are more rapid in megacities (17, 37).

### 4.2. Urban–sub-urban–rural variation in EMS supply and demand

The Tianjin Health Commission takes control of the overall planning and distribution of EMS resources at the city level in accordance with the population size of areas and the differences in district characteristics, such as the location, socioeconomic status, population density, age structure of the population, and health-seeking behaviors of patients. From urban areas to rural areas, population density and the level of economic development decrease gradually. The geographical area of the rural areas is larger and farther from the urban areas. To increase response rates, the emergency medical subcenter implemented regional ambulances dispatched in rural areas, which were different from urban and sub-urban areas in Tianjin.

Spatially, the EMS supply in Tianjin was the highest in urban areas, followed by sub-urban areas, and the lowest in rural areas. In particular, there were fewer EMS resources in Jinghai, Baodi, and Ninghe Districts. We should focus on strengthening EMS resources in these rural areas. The distribution of EMS resources was relatively fair according to the population and relatively unfair according to the geographical area and HRDI in Tianjin. This finding is consistent with the results of other studies showing that the fairness of health resource supply distribution according to the population was higher than that according to the geographical area in China (38, 39) mainly because the policies issued by the government aim mainly to focus on meeting the medical needs of the population (40).

We found that EMS demand was the highest in urban areas, followed by sub-urban areas, and the lowest in rural areas; this result is similar to other relevant research results (41), and EMS demand equity has significantly improved in Tianjin during the past 5 years. It has been reported that EMS use is fairly inequitable across groups, and patients in developed areas are more likely than those in less-developed areas to use EMS (24) possibly because although an ambulance is regarded as the best way for desperate patients to reach the nearest medical institutions, ambulance services are expensive and their cost cannot be entirely reimbursed by health insurance. The high cost of ambulance services may reduce the willingness to use EMS, especially for sub-urban and rural residents with lower incomes.

To optimize regionalized plans of EMS resources precisely, we explored differences in EMS demand characteristics. The time trends in the numbers of patients using EMS differed among urban, sub-urban, and rural areas. The EMS demand in urban areas was the highest throughout the study period. The annual growth rate of EMS appears to be higher among rural residents. This result indicated that the gap in EMS demand among urban, sub-urban, and rural areas was gradually narrowing (24, 40). As a result, EMS decision-makers should focus on additional approaches to EMS demand growth in sub-urban and rural areas in the coming years to meet demand on time. In addition, we found that the average age of patients in urban areas was older than those in sub-urban and rural areas; this difference may be related to the local age structure. Consequently, urban areas may have more elderly and critical EMS patients (42). Most disease diagnoses of EMS patients were non-specific diagnoses, followed by diagnoses of injuries, cerebrovascular diseases, and cardiovascular diseases. More patients with genitourinary system diseases were in urban areas; this result suggests that sub-urban and rural areas may have many neglected patients with genitourinary system diseases; more patients with infectious diseases were in sub-urban areas, possibly because of COVID-19 and because the airport and only designated hospital for COVID-19 treatment are located in sub-urban areas (13); compared to patients with other diseases, more patients with injuries, chemical–physical damage, and shock diseases seemed to be in the rural areas in Tianjin possibly because more highways are located in rural areas. The ability training of EMS staff should focus on training the ability to treat critical illnesses and these common diseases.

### 4.3. SDM pattern of EMS in urban, sub-urban, and rural areas

Interestingly, we observed that the high supply–high demand types with stationary demand trends were distributed mainly in urban areas; the low supply–high demand types with significant demand growth trends were distributed mainly in sub-urban areas; and the low supply–low demand types with the highest speed of demand growth were distributed mainly in rural areas. Our study comprehensively analyses urban–sub-urban–rural variation in EMS from both the supply and demand sides according to standardized electronic medical records for five consecutive years and uses classic measures and indicators (22, 24). The findings may improve the yield of emergency medical system reform in some upper-middle-income countries (43). On the one hand, the government should take control of overall planning and distribution to promote the reasonable supply and balanced distribution of high-quality healthcare resources, according to the distribution and service radius of medical emergency stations, residents' medical demand, population density, and other factors (24). In the emergency status, surrounding areas should provide peer-to-peer support to areas where emergencies occur and supplies are inadequate. On the other hand, the results illustrated that sub-urban and rural areas need more high-quality human resources and financial resources in the next few years (44). Decision-makers for EMS planning should further focus on three aspects: the spatial layout and optimal allocation of medical service facilities, the accessibility and equity of medical facilities, and the supply–demand matching of medical services, according to variations in EMS supply and demand characteristics among urban, sub-urban, and rural areas (27).

### 4.4. Limitations

This study also has several limitations. First, this study did not consider the spillover effects of adjacent units. Dispatching an ambulance follows the principle of proximity. For instance, if the patient with an EMS demand is on the border of one district, the station dispatching the ambulance may be dispatched from a station in another nearby district. Second, considering that doctors and nurses do not work at only one station and rotate at several stations in a subcenter, in turn, we analyzed only the spatial layout and equity of EMS supply according to the number of medical emergency stations. Third, we only described EMS demand from the perspective of utilization. Importantly, utilization is an objective measure of the throughput of a process relative to demand. Thus, we analyzed EMS demand using the data of EMS patients conservatively. Fourth, insights into urban–sub-urban–rural differences in EMS supply and demand from our study may be applicable to Tianjin, China only and might not be transferable to other regions with different population compositions and healthcare infrastructures.

## 5. Conclusion

Emergency medical services supply quantity and quality were promoted, and the supply met the demand in most districts in Tianjin after years of healthcare reform. The high supply–high demand types with stationary demand trends were distributed mainly in urban areas. The low supply–high demand types with significant demand growth trends were distributed mainly in sub-urban areas. The low supply–low demand types with the highest speed of demand growth was distributed mainly in rural areas. Our results may be a reasonable solution for improved EMS in Tianjin and other megacities in upper-middle-income countries.

## Data availability statement

The datasets presented in this article are not readily available because data and analyses are not publicly available as they were used under license for the current study. Data is however available from the authors upon reasonable request. Requests to access the datasets should be directed to caochunxia@tju.edu.cn.

## Ethics statement

The study protocol was approved by the Ethics Committee of Tianjin University (Project-No. 2021-166). As agreed by the Ethics Committee, this study was performed on anonymized health-related data; therefore, there was no need for written informed consent from individual patients (Federal Act on Research involving Human Beings, Art. 2).

## Author contributions

YL: conceptualization, methodology, and writing—original draft. JL and TL: data curation and data analysis. JG: resources and data curation. XL: visualization and data curation. HF: funding acquisition and supervision. CC: project administration, methodology, and writing—reviewing and editing. All authors contributed to the article and approved the submitted version.
