# Supplementary material for: Urban–sub-urban–rural variation in the supply and demand of emergency medical services
Source: Front Public Health. 2023 Jan 25;10:1064385. doi: 10.3389/fpubh.2022.1064385 (PMC9905235; doi:10.3389/fpubh.2022.1064385)
Supplement: Supplementary file 1 [file Data_Sheet_1.docx]

Supplementary Material

# Supplementary Figures and Tables

## Supplementary Tables

Supplemental Table 1 Diseases categories in the MPDS^†^

| **Categories** | **Diseases and related medical symptoms / signs** |
| --- | --- |
| Non-specific diagnoses | Unexplained headache, chest pain, dorsalgia, vertigo, lumbago, abdominal distention, palpitation, abdominal pain, vomiting, convulsion, jaundice, dyspnea, coma, hemoptysis, dizziness, blood urine, debilitation, paralysis, conscious disturbance, syncope, cough, hydrops, pain of limbs, chest distress, shock, electrolyte disturbance, respiratory arrest and fever; Sudden death; Clinical death; Multiple organ failure; Deep venous thrombosis; Myasthenia of limbs; Septicemia; Respiratory circulatory failure; Cardiac arrest. |
| Traumatic injury | Abdominal, trunk, extremities, chest, head, neck, lower back and multiple trauma; Bone trauma; Post-traumatic brain syndrome; Scalp hematoma. |
| Cardiovascular diseases | Trigeminy of ventricular prematurebeat; Idioventricular rhythm; Sustained ventricular tachycardia; Hypertensive sub-emergency; ventricular fibrillation; Sinus bradycardia; acute coronary syndrome; congenital heart disease; angina pectoris; chest complaint; old myocardial infarction; hypotension; sinus arrest; frequent premature ventricular contractions; hydropericardium; congestive heart failure; aortic dissection; viral myocarditis; hypertensive urgencies; chest distress; atrioventricular block; acute ST elevation myocardial infarction; acute non-ST elevation myocardial infarction; ventricular premature beat doublet; cardiomyopathy; Ι atrioventricular block; ΙΙΙ atrioventricular block; rheumatic heart disease; rhythmic rhythm; atrial flutter; ventricular flutter; sudden cardiac death; pre-excitation syndrome; Cardiac function class I / II /III / IV (NYHA classification); coronary disease; aentricular tachycardia; ventricular escape rhythm; serious cardiac arrhythmias; acute cardiac tamponade; pericarditis; palpitation; paroxysmal supraventricular tachycardia; Aymptomatic hypertension; Adams-Stokes syndrome; acute cardiac insufficiency; syncope; hypertensive disease; fast atrial fibrillation; Atrial fibrillation. |
| Cerebrovascular disease | Spontaneous subdural hematoma; Cerebral hemorrhage; Cerebral aneurysm; Cerebral embolism; Subarachnoid hemorrhage; cerebrovascular accident; Cerebrovascular accident sequelae; Transient ischemic attack; Cerebral infarction. |
| Respiratory diseases | Respiratory failure; Foreign body in air passage; Idiopathic pulmonary fibrosis; Pleurisy; Bronchial asthma; Dyspnea; Acute respiratory distress; Pneumothorax; Hyperventilation syndrome; Sleep apnea syndrome; Pulmonary embolism; Acute exacerbation of chronic bronchitis; Chronic obstructive pulmonary disease; Pulmonary encephalopathy; Pneumonia; Pulmonary heart disease; chest pain; lung abscess; hemoptysis; Pleural effusion; silicosis; Lung edema; Upper respiratory tract infection; Bronchiectasia; Spontaneous pneumothorax. |
| Digestive system diseases | Umbilical hernia; Drug-induced liver disease; Ulcerative colitis; Inguinal hernia; Cholelithiasis; Upper gastrointestinal bleeding; Hepatic cirrhosis; incisional hernia; Peritoneal effusion; Hepatic encephalopathy; Acute hemorrhagic enteritis; Acute gastritis; Incarcerated hernia; Gastric ulcer; Constipation; Liver abscess; Foreign body in stomach; Acute cholangitis; Acute cholecystitis; Acute appendicitis; Digestive tract perforation; Acute pancreatitis; Alcoholic cirrhosis; Peptic ulcer; jaundice; Intestinal obstruction; Acute hemorrhagic necrotizing pancreatitis; Duodenal ulcer; localized peritonitis; Diffuse peritonitis; Acute gastroenteritis; Lower gastrointestinal bleeding; vomiting, Femorocele; Other digestive diseases. |
| Poisoning | Chemical poisoning; Acute food poisoning; Pesticide intoxication; Drug poisoning. |
| Tumor | Gastric cancer; Carcinoma of pancreas; Colorectal carcinoma; Carcinoma of nasopharynx; Brain tumor; Osteosarcoma; Lung cancer; Trophoblastoma; Carcinoma of bladder; CCarcinoma of gallbladder; Colon carcinoma; Lymphoma; Breast cancer; Leukemia; Renal carcinoma; Esophageal carcinoma; Hysterocarcinoma; Bowel cancer; Liver cancer; Ovarian cancer; Glioma; Metastatic carcinoma; Other malignant / benign tumors. |
| Endocrine metabolic disease | Diabetes; Hypofunction crisis of the anterior pituitary gland; Diabetic hyperosmolar syndrome; Hypoglycemia; Hyperthyroidism; Pheochromocytoma; Ketoacidosis; Hypothyroidism crisis; Other endocrine metabolic diseases. |
| Exhaustion | Type II respiratory failure; Type I respiratory failure; Heart failure; Acute right ventricular failure; Chronic hepatic failure; Acute hepatic failure; Chronic respiratory failure; Chronic renal failure; Acute renal failure; Acute respiratory failure; Acute left ventricular failure; Heat exhaustion; Multiple organ failure. |
| Other nervous system diseases | Cephalitis meningica; aphasia; epilepsy; secondary epilepsy; seizures; guillain-barre syndrome; encephalitis; periodic paralysis; myasthenia gravis; transient ischemic attack; cataphora; neuralgia; paraplegina; parkinson's disease; hemiplegic paralysis; metabolic encephalopathy; intracranial infection. |
| Genitourinary system diseases | Hyperplasia of prostate gland; hematuria; uroschesis; cystitis; urinary tract infection; nephritis; uremia; nephrotic syndrome; urinary system stones; pyelonephritis; hydronephrosis; other urinary system diseases. |
| Obstetrics and gynecology diseases | Ovarian cyst torsion; hyperemesis gravidarum; functional uterine bleeding; eclampsia; preterm labor; Abortion; ectopic pregnancy; vaginal bleeding; dysmenorrhea; normal labor; placental abruption; puerperal fever; precipitate labor; placenta previa; Areeclampsia; amniotic fluid embolism; postpartum hemorrhage; rupture of uterus; pregnancy-induced hypertension; premature rupture of membranes; threatened abortion; other obstetrics and gynecology diseases. |
| Infectious disease | Syphilis; novel coronavirus vaccination response; tuberculous pleurisy, pulmonary tuberculosis, tuberculous meningitis, bone tuberculosis, spinal tuberculosis; rubella; influenza; epidemic hemorrhagic fever; rabies; meningococcal meningitis; coronavirus disease 2019, close contacts of corona virus disease 2019; viral encephalitis; bacillary dysentery; viral hepatitis; acute hemorrhagic conjunctivitis; acquired immune deficiency syndrome; epidemic parotitis; tetanus; bacillary phthisis varicella; hand foot and mouth disease; other infectious diseases. |
| Musculoskeletal and connective tissue diseases | Intervertebral disk hernia; rheumatoid arthritis; rhabdomyolysis; rhachitis; arthritis deformans; gout; scleroderma; femoral head necrosis; dermatomyositis; sciatica; osteoporosis; osteoarthritis; myelitis; lumbar disease; other musculoskeletal system and connective tissue disorders. |
| Ear; nose; throat and ophthalmology diseases | Foreign body in antrum auris; haemorrhagia nasalis; optic neuritis; acute epiglottitis; cataracta glauca; sudden acroisa; retinal detachment; cataract; other ear-nose-throat and ophthalmology diseases. |
| Pediatric diseases | Hyperpyrexia; febrile convulsion; neonatal jaundice; premature infant; febrile seizure; pneumonia of newborn; congenital heart disease; infantile diarrhea; neonatal respiratory distress syndrome; asphyxia of newborn; intussusception; intracranial hemorrhage of newborn; neonatal after resuscitation; low functioning of the newborn; normal newborn; other pediatric diseases. |
| Blood disease | Disseminated intravascular coagulation; leukemia emergency; aplastic anemia; purpura; anemia; hemolytic anemia; other blood diseases. |
| Psychobehavioral disorders | Mental and behavioral disorders; schizophrenia; manic psychosis; hysteria; mental anomaly; anxiety; neurasthenia; depression. |
| Chemical-physical damages | Adustum; snake bite, dog bite, cat scratch, poisonous insect bite, bee sting; acid burn; empyrosis; heat stroke; electrical burn; hanging oneself; alkali burn; heat cramps; electric shock; chilblain; inhalation injury; toxic gas inhalation; drowning; heat stroke. |
| Shock | Traumatic shock; Septic shock; Hemorrhagic shock; Anaphylactic shock; Cardiogenic shock; Hypovolemic shock. |
| Dermatogic diseases | Decubitus; lacerated wound; multiple skin and soft tissue injuries; erysipelas; multiple soft tissue injuries; cellulitis; skin and soft tissue infections. |
| Fluid and electrolyte imbalance | Respiratory acidosis; alkali poisoning; Acid poisoning; dehydration; respiratory alkalosis; hypercalcemia; hypokalemia; hyponatremia; hypocalcemia rheumatic contraction; hypocalcemia; hypernatremia; metabolic acidosis; diabetes insipidus; other fluid and electrolyte imbalance. |
| Immune system disease | Systemic lupus erythematosus; Laryngeal angioedema; Rheumatic fever; Drug eruption; Drug hypersensitivity; Infusion reaction; Sensitization dermatitis; Urticaria; Exfoliative dermatitis. |

^†^: Medical Priority Dispatch System

Supplemental Table2 The development state and EMS health resource supply of each district in Tianjin in 2021

| **Districts** | **Permanent Population^†^** | **Area^†^ (km^2^)** | **GDP^†^**  **(100 million yuan)** | **Number of medical emergency stations (per 100 000 people)** | **Number of medical emergency stations (per 1 km^2^)** | **HRDI** |
| --- | --- | --- | --- | --- | --- | --- |
| **Urban** |  |  |  |  |  |  |
| Nankai | 890 422 | 41 | 626 | 1.798 | 0.394 | 0.266 |
| Hexi | 822 174 | 37 | 990 | 1.703 | 0.378 | 0.254 |
| Hedong | 858 787 | 39 | 344 | 1.514 | 0.333 | 0.225 |
| Hebei | 647 707 | 27 | 365 | 1.853 | 0.444 | 0.287 |
| Hongqiao | 483 130 | 21 | 185 | 1.449 | 0.329 | 0.218 |
| Heping | 355 000 | 10 | 829 | 2.535 | 0.902 | 0.478 |
| **Suburban** |  |  |  |  |  |  |
| Xiqing | 1195 124 | 545 | 821 | 1.255 | 0.028 | 0.059 |
| Jinnan | 928 066 | 421 | 539 | 1.078 | 0.024 | 0.051 |
| Beichen | 909 643 | 478 | 624 | 1.539 | 0.029 | 0.067 |
| Dongli | 857 027 | 477 | 650 | 1.400 | 0.025 | 0.059 |
| **Rural** |  |  |  |  |  |  |
| Jizhou | 795 516 | 1 593 | 225 | 2.138 | 0.011 | 0.048 |
| Wuqing | 115 1313 | 1 574 | 816 | 1.216 | 0.009 | 0.033 |
| Jinghai | 787 106 | 1 476 | 433 | 1.270 | 0.007 | 0.029 |
| Baodi | 722 367 | 1 523 | 354 | 1.523 | 0.007 | 0.033 |
| Ninghe | 395 314 | 1 414 | 313 | 2.276 | 0.006 | 0.038 |
| Binhai | 2 067 318 | 2 270 | 5 871 | 1.790 | 0.016 | 0.054 |

**^†^**: Data from Tianjin Bureau of Statistics; GDP: Gross domestic product; HRDI: Health resource density index.

Supplemental Table 3 The equity in EMS health resource demand in Tianjin

| **Year** | **G_p_** | **G_g_** | **G_h_** |
| --- | --- | --- | --- |
| 2017 | 0.481 | 0.657 | 0.601 |
| 2018 | 0.474 | 0.653 | 0.598 |
| 2019 | 0.434 | 0.648 | 0.583 |
| 2020 | 0.318 | 0.638 | 0.549 |
| 2021 | 0.231 | 0.634 | 0.522 |

G_p_: the G values of EMS health resource supply by population; G_g_: the G values of EMS health resource supply by geographical distribution; G_h_: the G values of EMS health resource supply by HRDI.

## Supplemental Figures

**
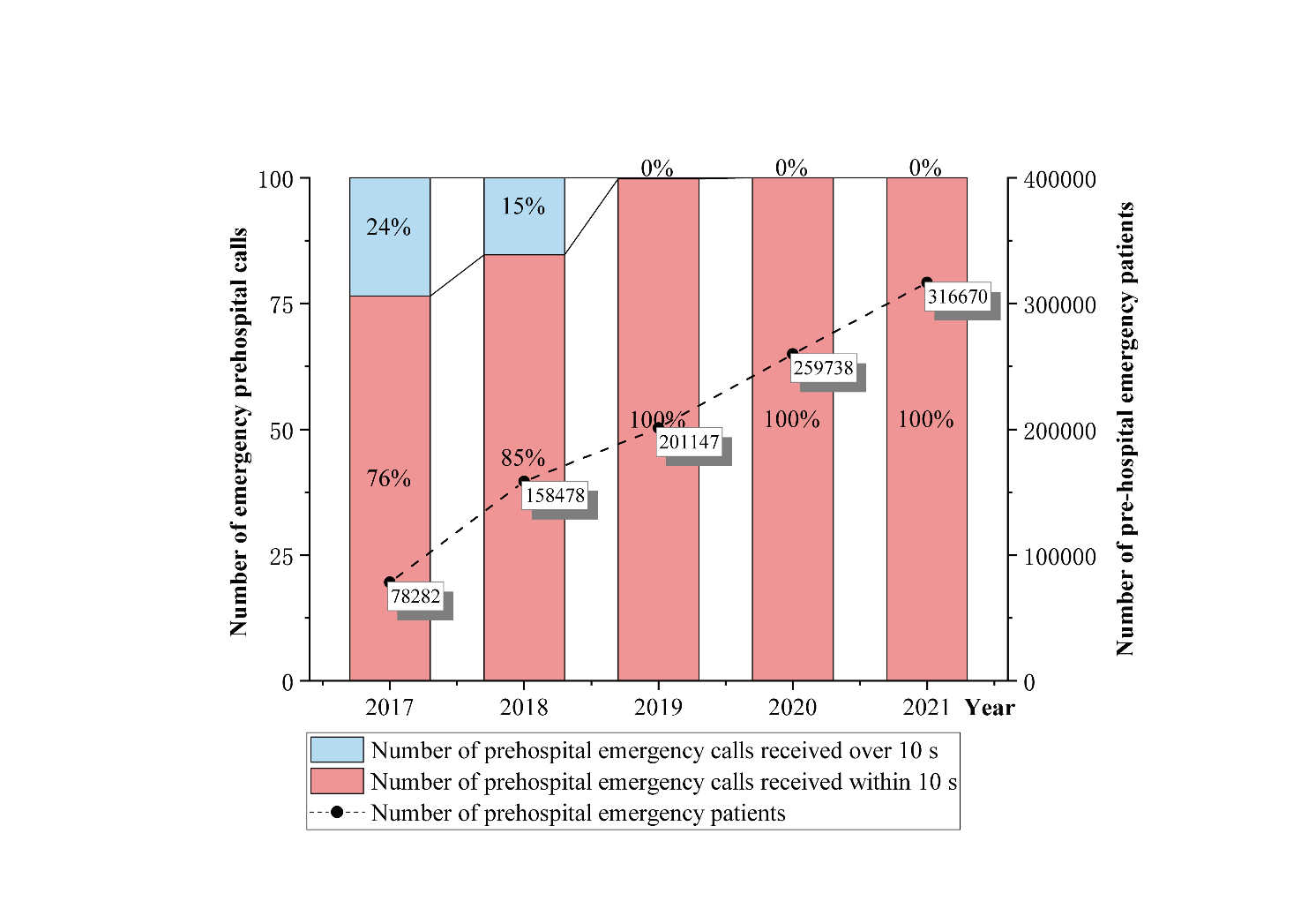
**

**Supplemental Figure 1** Prehospital emergency calls and patients in Tianjin, China


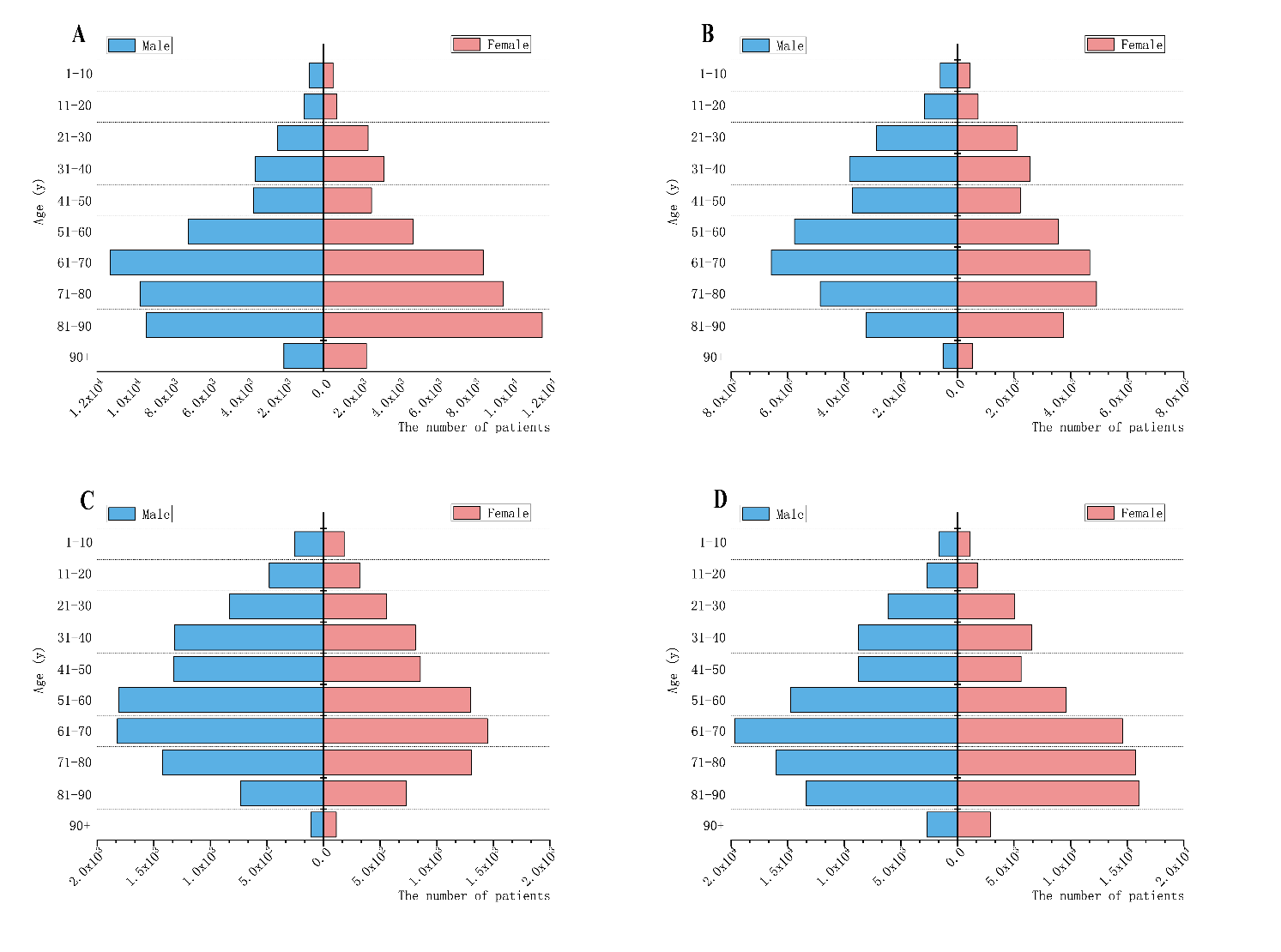


**Supplemental Figure 2** Gender and age of patients using the EMS per year in Tianjin

(A) Urban areas; (B) suburban areas; (C) Rural areas; (D) Total.
